# Multiparametric characterization of white matter alterations in early stage Huntington disease

**DOI:** 10.1038/s41598-021-92532-1

**Published:** 2021-06-23

**Authors:** Isaac M. Adanyeguh, Francesca Branzoli, Cécile Delorme, Aurélie Méneret, Marie-Lorraine Monin, Marie-Pierre Luton, Alexandra Durr, Emanoel Sabidussi, Fanny Mochel

**Affiliations:** 1 INSERM U 1127, CNRS UMR 7225, Sorbonne Universités, UPMC Univ Paris 06 UMR S 1127, Institut du Cerveau Et de La Moelle Épinière, ICM, 75013 Paris, France; 2 Center for NeuroImaging Research (CENIR), Institut du Cerveau Et de La Moelle Épinière, 75013 Paris, France; 3 Department of Neurology, AP-HP, Pitié-Salpêtrière University Hospital, Paris, France; 4 Department of Genetics, Center for Neurometabolic Diseases, AP-HP, La Pitié-Salpêtrière University Hospital, 47 Boulevard de l’Hôpital, 75013 Paris, France

**Keywords:** Biomarkers, Huntington's disease, Biomarkers

## Abstract

Huntington’s disease (HD) is a monogenic, fully penetrant neurodegenerative disorder. Widespread white matter damage affects the brain of patients with HD at very early stages of the disease. Fixel-based analysis (FBA) is a novel method to investigate the contribution of individual crossing fibers to the white matter damage and to detect possible alterations in both fiber density and fiber-bundle morphology. Diffusion-weighted magnetic resonance spectroscopy (DW-MRS), on the other hand, quantifies the motion of brain metabolites in vivo, thus enabling the investigation of microstructural alteration of specific cell populations. The aim of this study was to identify novel specific microstructural imaging markers of white matter degeneration in HD, by combining FBA and DW-MRS. Twenty patients at an early stage of HD and 20 healthy controls were recruited in a monocentric study. Using diffusion imaging we observed alterations to the brain microstructure and their morphology in patients with HD. Furthermore, FBA revealed specific fiber populations that were affected by the disease. Moreover, the mean diffusivity of the intra-axonal metabolite *N*-acetylaspartate, co-measured with *N*-acetylaspartylglutamate (tNAA), was significantly reduced in the corpus callosum of patients compared to controls. FBA and DW-MRS of tNAA provided more specific information about the biological mechanisms underlying HD and showed promise for early investigation of white matter degeneration in HD.

## Introduction

Huntington disease (HD) is a rare and severe autosomal dominant neurodegenerative disorder caused by an expansion of cytosine–adenine–guanine trinucleotide (CAG) repeats in the Huntingtin (*HTT*) gene^1^. One of the most targeted brain abnormalities in HD is gray matter atrophy, which mostly involves subcortical gray matter structures, but also several cortical regions including frontal and parietal lobes^2,3^. In the last decade, several studies employing either structural or diffusion magnetic resonance imaging (MRI) have underscored the role of white matter (WM) degeneration in the pathophysiology of HD, showing evidence of widespread WM atrophy^4^, as well as abnormalities in several WM pathways, including striatal projection fibers and the corpus callosum^5–7^.

Diffusion MRI enables the investigation of WM microstructural alterations and provides useful insights into the pathological processes underlying brain atrophy. Diffusion tensor imaging (DTI) has been widely used to explore WM microstructural changes in HD and alterations have been observed in several brain regions including the corpus callosum, corona radiata, internal and external capsules, thalamic radiations, corticospinal tract, the cingulate gyrus and longitudinal fasciculus^5,8–11^. Most studies have reported reduced fractional anisotropy (FA) and elevated mean diffusivity (MD) in the WM of patients with HD compared to controls, suggesting axonal degeneration. Loss of axonal integrity has been linked with clinical disability and has been proposed to represent an early marker of neurodegeneration in HD^12,13^. DTI metrics are nevertheless non-specific markers of WM degeneration and do not allow the identification of precise mechanisms of the underlying axonal pathology. In particular, DTI suffers from the confounding effect of crossing fibers, which may artificially reduce FA values. In addition, DTI does not enable the separation between intra- and extra-cellular water components; therefore, the observed changes cannot be attributed to specific biological processes^14^. Given that up to 90% of WM voxels may contain more than one fiber population^15^, assessing both microscopic fiber density and macroscopic changes in fiber bundle morphology can provide additional insights on the processes contributing to WM degeneration^16^. Fixel-based analysis (FBA) is a novel whole-brain higher-order model of fiber estimation that incorporates the constrained spherical deconvolution (CSD) approach to compare specific fiber population in a voxel, known as fixel^16^. Alterations in tissue microstructure evaluated with FBA can be attributed to a reduced amount of intra-axonal volume (fiber density, FD), to a similar fiber density but reduced total area occupied by the axons (fiber-bundle cross-section, FC), or to a combination of both processes (fiber density and cross-section, FDC)^16^. This method thus overcomes the limitations associated with other diffusion MRI approaches that cannot differentiate between multiple fiber populations in a single voxel^15,17^, which is of crucial relevance especially in brain regions with crossing fibers. Remarkably, a previous study recently showed that FBA metrics may outperform DTI metrics in spinocerebellar ataxia, another group of polyglutamine disorders^18^.

A complementary approach to diffusion MRI for the investigation of WM alterations is diffusion-weighted MR spectroscopy (DW-MRS). DW-MRS is a promising technique that allows measuring the diffusivity of several intra-cellular metabolites *in vivo*^19,20^. Thanks to the specific compartmentalization of brain metabolites in different cell types, this method provides information on microstructural and metabolic alterations of specific cell populations in brain tissue, notably neurons and glia, without confounding effects from the extra-cellular space. In particular, the diffusion of the intra-neuronal metabolite *N*-acetylaspartate was suggested as a marker of intra-axonal damage before irreversible loss in multiple sclerosis^21^.

In this study, we aimed at investigating WM degeneration in HD by combining two novel approaches for tissue microstructural characterization. For this purpose, we employed FBA at 3 T in a cohort of 20 patients with HD, compared with 20 age- and gender-matched healthy controls. Furthermore, because the corpus callosum was suggested to be one of the WM regions that is affected earlier in HD^22^, we quantified metabolite diffusivity in this region using DW-MRS.

## Results

### Alterations in DTI metrics

**Figure 1:**
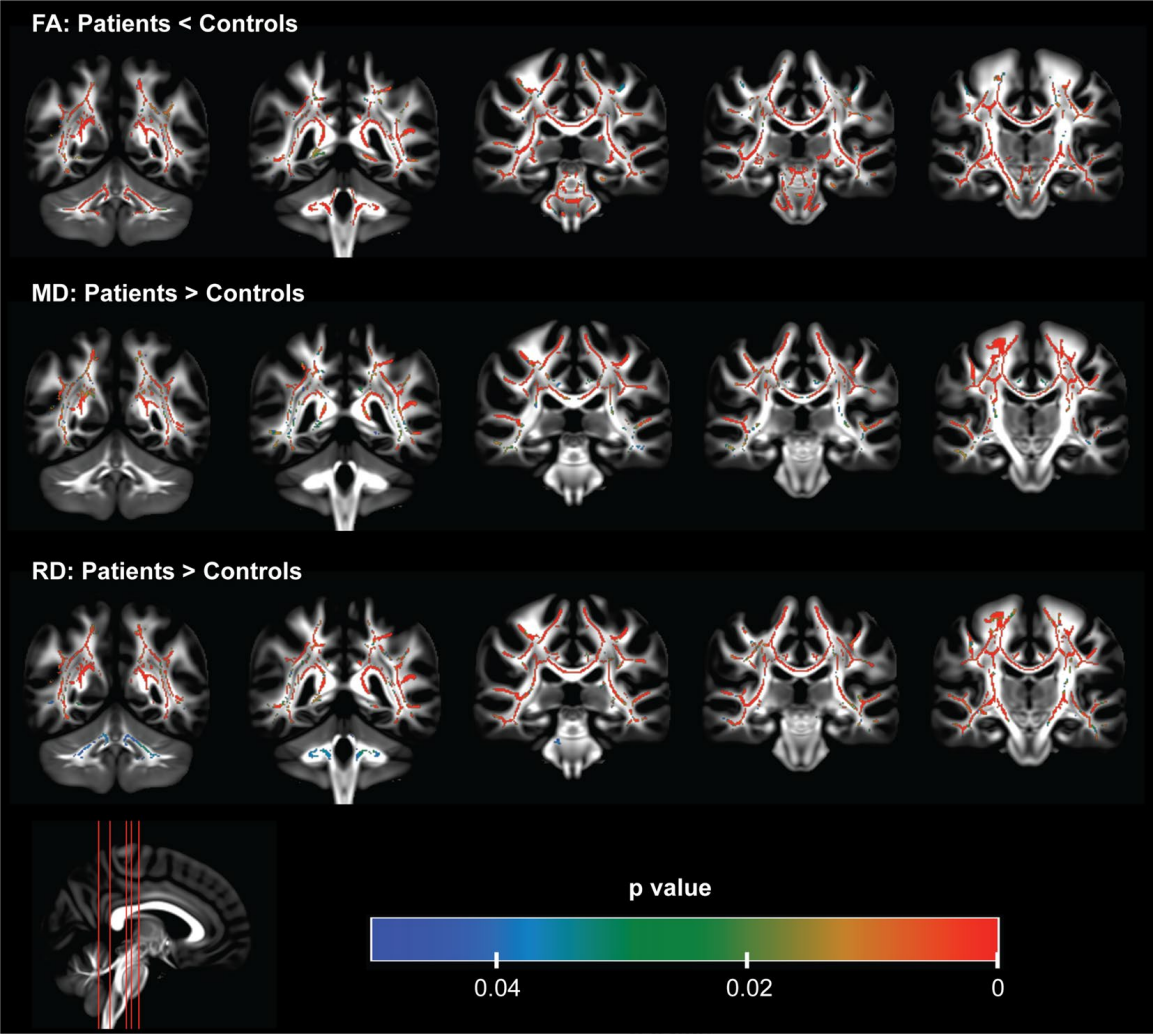
Diffusion tensor analysis showing altered brain white matter microstructure in patients with HD using tract-based spatial statistics. Reduced fractional anisotropy (FA), increased mean diffusivity (MD) and increased radial diffusivity (RD) were observed in the corpus callosum, sagittal stratum, thalamic radiation bundle, superior longitudinal fasciculus, external and internal capsules, corona radiata, and the cingulum. FA was also reduced in the corticospinal tract, and pontine crossing tract. FA and RD were also altered in the cerebellar and cerebral peduncles. The color bar represents significant regions (p < 0.05) of alterations in DTI metrics.

Diffusion data was not acquired for one patient with HD due to important motion artifact during data acquisition. The average motion and total outliers were comparable between patient and control groups (Supplementary Fig. 1). TBSS analyses of DTI metrics showed patterns of altered microstructure in the brain of patients with HD. Compared to controls, patients showed significantly (p < 0.05) reduced FA and increased MD and RD in several brain regions including the corpus callosum, corticospinal tract, pontine crossing and cerebellar peduncles (Fig. 1). These alterations significantly (p < 0.05) correlated with clinical metrics – UHDRS and CAP (Table 1).

**Table 1:** Correlation of DTI and FBA metrics to clinical scores.

Region	FA		MD		RD		FC		FDC	
	r	p	r	p	r	p	r	p	r	p
**UHDRS**										
CC	− 0.82	**0.001**	0.82	** < 0.001**	0.85	** < 0.001**	–	–	− 0.56	0.212
CR	− 0.52	0.202	0.68	**0.019**	0.66	**0.036**	− 0.28	1.44	− 0.45	0.322
PTR	− 0.67	**0.039**	0.63	**0.037**	0.72	**0.012**	− 0.09	0.727	–	–
SST	− 0.78	**0.020**	0.37	0.153	0.62	0.054	− 0.45	0.631	− 0.65	0.071
EC	− 0.84	**0.001**	0.68	**0.021**	0.81	**0.001**	− 0.67	**0.044**	− 0.74	**0.011**
Cing	− 0.73	**0.015**	0.46	0.150	0.66	**0.005**	–	–	− 0.52	0.235
Fx_ST	− 0.87	** < 0.001**	–	–	0.8	**0.002**	–	–	− 0.62	0.102
SLF	− 0.72	**0.002**	0.77	**0.003**	0.8	**0.002**	–	–	–	–
**CAP**										
CP	− 0.69	**0.037**	–	–	0.35	1.419	− 0.52	0.223	− 0.65	**0.046**
PCT	− 0.56	0.244	–	–	–	–	− 0.38	0.283	− 0.53	0.205
mLEM	− 0.69	**0.039**	–	–	0.47	0.646	− 0.62	0.089	− 0.76	**0.006**
IC	− 0.15	1.152	0.25	1.741	0.24	1.473	− 0.74	**0.010**	− 0.70	**0.020**
SST	− 0.40	0.766	− 0.10	0.704	0.13	0.636	− 0.61	0.080	− 0.71	**0.019**
EC	− 0.60	0.164	0.36	1.161	0.50	0.507	− 0.77	**0.005**	− 0.74	**0.010**
Fx_ST	− 0.70	**0.032**	–	–	0.63	0.110	–	–	− 0.80	**0.002**

Dashes (–) represent regions that were not selected since they showed no differences between patients and controls. CC: corpus callosum, CR: corona radiate, PTR: posterior thalamic radiation, SST: sagittal stratum, EC: external capsule, Cing: cingulum, Fx_ST: fornix/stria terminalis, SLF: superior longitudinal fasciculus, CP: cerebellar peduncle, PCT: pontine crossing tract, mLEM: medial lemniscus, IC: internal capsule. P values have been corrected for multiple comparisons using the holm-bonferroni step-down correction.

### Fixel-specific characterization

**Figure 2:**
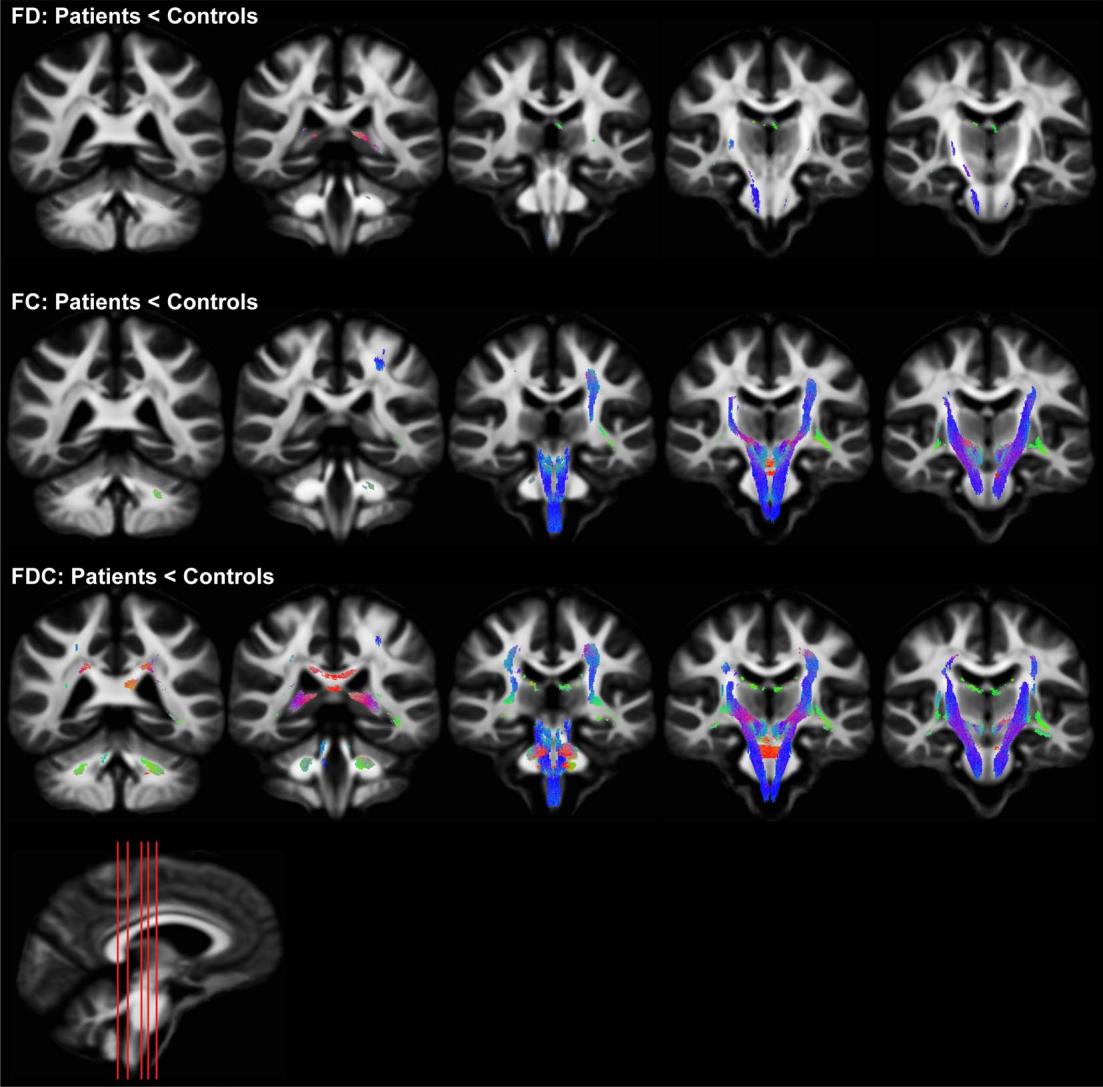
Fixel-based analysis showing significantly reduced fiber density (FD), fiber bundle cross-section (FC) and fiber density and cross-section (FDC) in patients with HD. Reduction in FD was limited to the corticospinal tract while FC and FDC were significantly reduced in the corticospinal tract, cerebellar peduncles, pontine crossing tracts, internal and external capsules, medial lemniscus, cerebral peduncles, sagittal stratum, and corona radiata. FDC was also reduced in the fornix/stria terminalis and the corpus callosum in patients with HD. Significantly reduced streamlines are directionally colored (red: left–right, green: anterior–posterior, blue: superior-inferior).

Compared to controls, patients showed significantly (p < 0.05) reduced fiber density especially in the corticospinal tract (Fig. 2). While fiber density changes were limited in patients with HD, FC and FDC were significantly reduced in the corticospinal tract, cerebellar peduncles, pontine crossing tracts, internal and external capsules, medial lemniscus, cerebral peduncles, sagittal stratum, and corona radiata (Fig. 2). Furthermore, patients with HD displayed decreased FDC in the fornix/stria terminalis and the corpus callosum. Moreover, FC and FDC showed significant (p < 0.05) correlations with clinical metrics – UHDRS and CAP (Table 1).

### Metabolite diffusion

**Figure 3:**
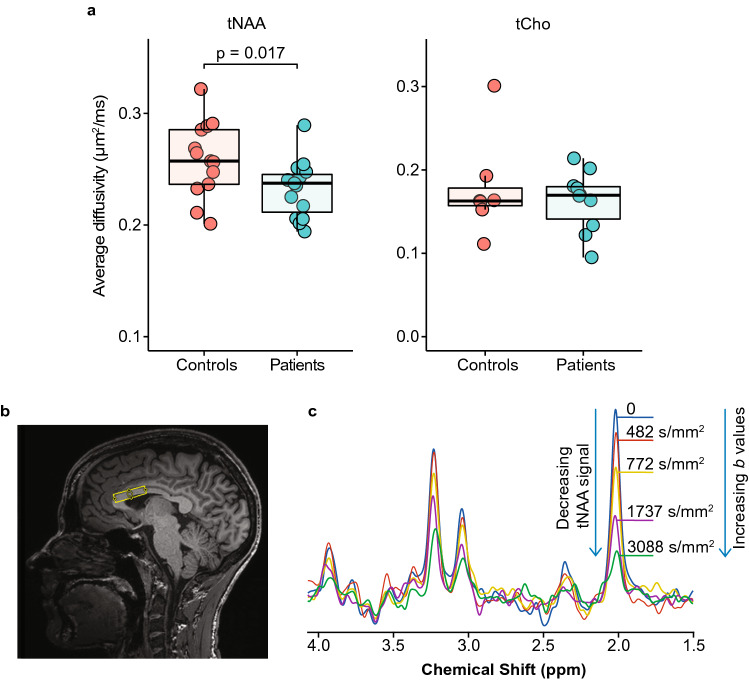
Diffusivity of metabolites and the representation of diffusion-weighted magnetic resonance spectroscopy (DW-MRS) acquisition. (**a**) Average diffusivity of total *N*-acetylaspartate (tNAA) and total choline (tCho) between patients and controls. Significantly lower average diffusivity of tNAA was observed in patients with HD while no difference in diffusivity was observed in tCho. (**b**) Position of the 15 × 32 × 8 mm^3^ voxel for DW-MRS acquisitions in the corpus callosum. (**c**) Signal attenuation in relation to an increase in the *b* values used in DW-MRS acquisitions.

The average diffusivity of tNAA was reported in 13 controls and 15 patients, while the average diffusivity of tCho was reported in 7 controls and 10 patients. The average diffusivity of tNAA was significantly lower (p = 0.017) in the corpus callosum of patients with HD compared to healthy controls (Fig. 3a). There was no significant difference in the average diffusivity of tCho (Fig. 3a). Average tNAA diffusivity correlated negatively with CAG length (r = -0.505, p = 0.046) but did not correlate with the DTI and FBA metrics extracted from the corpus callosum. Average diffusivity of tCr was not reported since more than half of the data did not pass the quality check determined by the Cramér–Rao lower bounds estimated errors of metabolite quantification.

## Discussion

In order to achieve a specific in vivo characterization of WM axonal pathology in patients with HD, we applied for the first time in this disease two innovative methods – FBA and DW-MRS. FBA allowed us to explore alterations in individual WM fiber populations within the imaging voxels, and revealed significant reductions in the fiber density (FD), fiber-bundle cross-section (FC) and the fiber density and cross-section (FDC) metrics in several WM regions, including the corpus callosum, of patients with HD compared to controls. On the other hand, DW-MRS enabled the evaluation of cell-specific intra-cellular damage contributing to WM degeneration in HD by quantifying the diffusion of brain metabolites in intra-cellular compartments. Using DW-MRS, we observed significantly lower tNAA diffusivity in the corpus callosum of HD patients, suggesting early axonal alterations in this region.

In this study, we focused on WM microstructural alterations in HD, as WM degeneration processes have been suggested as already on-going in premanifest HD subjects^22^. In particular, the corpus callosum has been proposed to play a crucial role in HD disability as the primary cortical projection system connecting the two hemispheres^23^. Callosal microstructural abnormalities were observed in premanifest carriers and early symptomatic HD using both DTI and higher order diffusion models^22^, and these changes were correlated with clinical measures, thus pointing out the role of disrupted inter-hemispheric connection in the clinical disability in HD. However, the precise biological mechanisms of WM degeneration are still not fully understood.

Decrease in FA and increase in MD and RD have been reported in patients with HD compared to controls in several WM regions, including the corpus callosum^5,8,11^. Our DTI results are therefore in line with previous reports. Differences in DTI metrics may reflect several pathological abnormalities, such as demyelination, reduction in the number of axons, alterations in fiber organization, or a combination of different processes. The major disadvantage of DTI however is that it is prone to some level of uncertainty in its biological interpretation^17^. Also, the simplistic unimodal Gaussian approximation, which assumes a single fiber bundle in a voxel, presents several pitfalls such as stochastic and deterministic errors when it is used to reconstruct the fibers pathways^17^, making it difficult to attribute a specific biological meaning to the observed changes. Advanced methods based on higher-order modeling allow for a more specific characterization of WM microstructure. Among these, neurite orientation dispersion and density imaging (NODDI)^24^ provides information on fiber density and dispersion, as well as free water component, within the imaging voxel, reducing the confounding effects associated with atrophy-related changes in extra-cellular compartments. Using the NODDI approach, reduced axonal density was recently observed in several WM regions of premanifest HD subjects compared to controls and these alterations were found to correlate with measures of disease progression in the corpus callosum^22^. However, results obtained using NODDI do not include information related to specific fiber populations within a voxel. In other words, it shares similar limitations as DTI.

In contrast, the CSD algorithm^25,26^ that is incorporated in the FBA pipeline accounts for non-WM tissue and allows the extraction of multiple fibers and their orientations. With this method, interconnected fixels are identified to show clusters of fiber pathways that are significantly different between the populations under investigation. In effect, FBA attributes observed changes to specific fiber populations within a voxel and it does so by describing the changes within a voxel and across voxels. In this study, FBA showed decrease in FD, FC and FDC in several regions in patients with HD, particularly in the corticospinal tract (CST). The CST is the pathway that is linked to the primary motor cortex. Since motor impairment is one of the characteristics of HD, it stands to reason that this pathway is greatly affected in patients with HD. Interestingly, our results suggest that our cohort may not be that different from controls in terms of fiber density (intra-axonal volume). This could be due to the fact that our cohort is at the early stage of the disease. Our results also show that the fiber bundle morphology is the main driving force of the pathology. However, in combining the fiber density and the fiber morphology information we observe the largest decrease in fibers in patients with HD. Thus, axonal loss as well as fiber bundle atrophy work hand in hand in the HD pathology. FDC is thus suggested as the most robust and useful metric to investigate neurodegeneration and the status of the remaining white matter tissue before irreversible loss, given the tight interplay between fiber density reduction and atrophic processes in neurodegenerative diseases^16^. Noticeably, we observed strong correlations between diffusion and clinical metrics.

While standard diffusion imaging investigates the diffusivity of water, DW-MRS allows the quantification of the diffusivity of intra-cellular metabolites, providing microstructural information on specific cellular compartments – mostly neurons and glia – without confounding effects from the extra-cellular milieu. DW-MRS has been applied to several pathologies to explore altered metabolite diffusivity in the brain of patients with mitochondrial disorders^27^, multiple sclerosis^21,28^, neuropsychiatric systemic lupus erythematosus^29^, and aging healthy adults^30^. DW-MRS of the neuronal metabolite tNAA allows probing the intra-axonal integrity before irreversible neuronal loss^21^ and may therefore be a useful marker of early axonal damage in HD. In the present study, we observed reduced average tNAA diffusivity in the corpus callosum of patients with HD, likely reflecting intra-axonal damage. This observation is in good agreement with findings from the diffusion analysis of the corpus callosum (i.e., reduced FDC in patients with HD), which also provided evidence of neuronal damage in this region. In contrast, we did not observe a significant change in the diffusivity of the glial marker tCho. However, due to low SNR, several subjects were excluded from the analysis of tCho data, which may have reduced the statistical power of this measure below the detectability threshold^31^. Therefore, from our data we could not rule out a contribution of inflammation-related glial cell alterations to the pathological changes occurring in the corpus callosum of HD patients. While DW-MRS remains a powerful tool to probe metabolite diffusivities, it is impacted by low metabolite SNR and thus requires acquisition of several spectra over a long acquisition time, which is challenging in patients with movement disorders like HD.

In conclusion, we corroborated the presence of WM abnormalities at early stages of HD. While DTI was confirmed as a useful and sensitive tool to detect microstructural alterations in brain tissue, FBA showed promise as a robust approach enabling specific biological interpretation of the diffusion metrics. Furthermore, DW-MRS provided complementary microstructural information by probing metabolite diffusion in specific intra-cellular compartments. Lower tNAA diffusion in HD was compatible with the presence of intra-axonal damage in the corpus callosum and consistent with FBA findings. As tNAA diffusion probes the intra-axonal damage that precedes neuronal loss, future studies shall aim at detecting tNAA diffusion alterations in premanifest HD subjects, prior to measurable atrophy.

## Materials and methods

### Recruitment of participants and clinical evaluation

The local ethical committee (CPP Ile de France VI) approved this observational study (NCT02639871) promoted by the “Assistance Publique des Hôpitaux de Paris”. All methods were performed in accordance with the relevant guidelines and regulations. Participants over 18 years of age were enrolled after they signed a written informed consent and had no contraindications to magnetic resonance examinations, history of severe head injury, or participating in another trial. Furthermore, patients who were included had CAG repeat length equal to or greater than 39 repeats and had a motor score on the UHDRS (unified Huntington disease rating scale)^32^ less than 40. In addition, subjects who were being treated with tetrabenazine, pregnant, breastfeeding or unable to understand the information in the consent form were not included in the study. Twenty healthy individuals (9 men and 11 women; mean age 42.2 years ± 12.6) and twenty patients with HD (9 men and 11 women; mean age 45.5 years ± 6.8) were recruited for the study (Table 2). The UHDRS was used to clinically assess the motor capabilities of subjects. Furthermore, the CAG-age product (CAP) was used to assess disease burden, defining an age-normalized measure of HD severity^33^.

**Table 2:** Demographic parameters of recruited participants.

	Controls	HD patients	p value
Number	20	20	–
Men/women	9/11	9/11	1.000
Age (years)	42.15 ± 12.60 [36.25–48.05]	45.50 ± 6.83 [42.30–48.70]	0.305
Body Mass Index (kg/m^2^)	23.97 ± 3.42 [22.37–25.57]	23.37 ± 3.14 [21.91–24.84]	0.569
UHDRS (max bad /124)	1.20 ± 1.06 [0.71–1.69]	12.50 ± 7.34 [9.07–15.93]	< 0.001
Pathological CAG repeat expansion in *HTT* gene	–	44.85 ± 4.09 [42.93–46.77]	–
CAP (CAG age product)	–	493.55 ± 120.11 [437.34–549.76]	–

Data are presented as mean ± standard deviation [95% confidence interval]. P values were calculated using Welch ANOVA and significance was set at alpha < 0.05. Differences in gender were calculated using chi-square analysis.

### Imaging and MRS protocols

All magnetic resonance acquisitions were carried out on a 3 T whole-body Siemens Magnetom Prisma scanner (Siemens Medical Solutions, Erlangen, Germany) with a standard Siemens transmit body coil and 64-channel receive head-neck coil array. A three-dimensional T_1_-weighted image (*T*_*R*_: 2300 ms, *T*_*E*_: 4.18 ms, *T*_*I*_: 900 ms, field-of-view (FOV): 256 × 240 mm^2^, slice thickness: 1 mm) was acquired to allow for positioning of the volume of interest (VOI) and for volumetric analysis. Diffusion-weighted 2D spin-echo echo planar imaging (*b* values: 2500 s/mm^2^ (60 directions), 900 s/mm^2^ (32 directions) and 300 s/mm^2^ (8 directions), FOV: 208 × 208 mm^2^, *T*_*E*_: 71 ms, *T*_*R*_: 2910 ms, slice thickness: 2 mm, multiband acceleration factor: 3) was acquired to evaluate the brain microstructure in HD. Similar acquisitions were performed but with opposite phase-encode blip for distortion correction. The diffusion acquisitions were interleaved with several non-diffusion-weighted reference images for motion correction.

DW-MRS was performed in a 15 × 32 × 8 mm^3^ VOI positioned on the anterior part of the corpus callosum (Fig. 3b) using a semi-localized by adiabatic selective refocusing (sLASER) sequence coupled with bipolar diffusion weighting gradients (*T*_*E*_: 120 ms, *T*_*R*_: 3000 ms, gradient duration: 18 ms)^34^. Diffusion weighting was applied in two orthogonal directions that were parallel (*b* values: 0, 482, 772, 1737 and 3088 s/mm^2^, 48 spectra per *b* value) and perpendicular (*b* values: 0, 964, 1544, 3474, 6176 s/mm^2^, 48 spectra per *b* value) to the callosal fibers. FAST(EST)MAP^35^ was used to perform B_0_ shimming in the VOI. The variable pulse power and optimized relaxation delays^36^ were used for water suppression after determining the power needed for maximum water suppression. A reference scan, with no water suppression, was acquired for each *b* value and direction and used for eddy current correction. Cardiac gating was performed for all DW-MRS acquisitions using a peripheral pulse unit to reduce signal fluctuations induced by physiological motion.

### Post-processing and analysis

#### Diffusion image analysis

Only the diffusion data acquired with *b* value of 2500 s/mm^2^ was used to estimate microstructural alterations using DTI and FBA methods. This was done to improve the estimation of the fiber orientation distribution function (Supplementary Fig. 2) and limit the contribution of extra-axonal signal present at low *b* values during the estimation of FBA metrics. Diffusion-weighted MRI data were processed using MRtrix3Tissue version 5.2.9 (https://3Tissue.github.io), a fork of MRtrix3, and FSL version 6.01 tools. Though HD is a movement disorder, none of the data failed the initial visual inspection. Furthermore, we used the FSL quality assessment tool^37^ to estimate the absolute motion within the patient and control groups in order to account for the bias due to excessive motion in either group. Noise in the data was first removed using a principal component analysis^38^ and resized by a factor of 2 to improve the contrast-to-noise ratio. Susceptibility and eddy current distortions caused by the fast-changing gradients as well as motion artefacts from participants were corrected using FSL eddy and topup^39,40^. Furthermore, the data were corrected for field inhomogeneity and the image intensity was normalized across all subjects to reduce bias.

#### Diffusion tensor imaging (DTI) analysis

The pre-processed data were fitted with the diffusion tensor model to extract DTI maps that included the fractional anisotropy (FA, a measure that reflects structural damage), radial diffusivity (RD, a measure of diffusion across axons that may signify damage to the myelin), mean diffusivity (MD, total amount of diffusion within a voxel), and axial diffusivity (AD, often considered the diffusion along the principal axis).

#### Fixel-based analysis (FBA)

Estimation of multiple fibers within a voxel using FBA provides a way to extract complex white matter pathways, which are otherwise not properly estimated using the diffusion tensor model. An unsupervised estimation of the response function that did not require segmentation of the T_1_ image was used to estimate the white matter, gray matter and cerebrospinal fluid response functions^25^. The single-shell 3-tissue CSD method^26^ was used to estimate the fiber orientation distribution (FOD) of white matter, gray matter and cerebrospinal fluid from the average of all response functions. This method allowed the minimization of partial volume effect from the non-white matter compartments – gray matter and cerebrospinal fluid. The FODs were then registered to a template that was created from the FOD of 10 controls and 10 patients and then processed to generate an estimation of the intra-axonal compartment volume that can be linked to FD, FC, or a combination of both processes (fiber density and cross-section, FDC)^16^. A whole brain fiber tract map of the template image was generated with 20 million tracts after which it was filtered^41^ to 2 million tracts to reduce biases and generate an anatomically meaningful whole brain tract map.

#### Metabolite apparent diffusion coefficient

DW-MRS data were pre-processed using in-house scripts written in MATLAB release R2016 (Mathworks, Natick, MA, USA). Shot-to-shot phase and frequency corrections were performed using the tNAA peak^42^. The non-water suppressed spectra were used for eddy current corrections. The spectra were then averaged for each *b* value and quantified with LCModel, using a simulated basis set. A Cramér–Rao lower bounds estimated errors of metabolite quantification of ≤ 20% was used to select reliably quantified metabolites. The sequential increase in *b* values during acquisition lead to the attenuation of the metabolite signals (Fig. 3c) and hence a decrease in the signal-to-noise ratio (SNR). Therefore, only the most prominent metabolites – tNAA, total choline (tCho) and total creatine (tCr) – were analyzed. The parallel and perpendicular diffusivities were calculated by performing linear fits of the natural logarithm of metabolite signal decays at different *b* values, in the two directions. The average diffusivity of the metabolites of interest was calculated by averaging parallel and perpendicular diffusivities.

### Statistical analyses

Two-tailed tests were performed for all statistical analyses. Demographic parameters were compared with Welch ANOVA (body mass index, age and UHDRS scores) and Chi-Square (gender) to identify differences between controls and patients with HD. The 95% confidence interval was also determined for all demographic parameters. Differences in average diffusivity of metabolites between patients and controls were evaluated using analysis of covariance while controlling for age, followed by a Bonferonni correction. Tract-based spatial statistics (TBSS)^43^ and FSL randomize with 5000 permutations were used to make voxel-wise comparisons of the DTI maps between controls and patients with HD with family-wise error corrected significance at alpha = 0.05. The mean of each region that showed significant differences was extracted using the John Hopkins University white matter atlas in order to perform correlation analysis with clinical parameters. Furthermore, the threshold-free cluster connectivity-based fixel enhancement^44^ was used to test for significant differences of FD, FC and FDC between controls and patients with HD with family-wise error corrected significance at alpha = 0.05. Mean fixel values from regions of significance were also extracted for correlation analyses. Pearson correlation was used to find the relation between imaging and spectroscopy parameters to clinical scores. When significant, Holm-Bonferroni^45^ approach was used to correct for multiple testing.

## Supplementary Information

Supplementary Information.

## Data Availability

The datasets analyzed for this study are not publicly available because an extension of the study is still ongoing. Requests to access the datasets should be directed to [Fanny Mochel, fanny.mochel@upmc.fr].
