# Supplementary material for: Multiparametric characterization of white matter alterations in early stage Huntington disease
Source: Sci Rep. 2021 Jun 23;11:13101. doi: 10.1038/s41598-021-92532-1 (PMC8222368; doi:10.1038/s41598-021-92532-1)
Supplement: Supplementary file 1 — Supplementary Information. [file 41598_2021_92532_MOESM1_ESM.docx]

**Supplementary Figure 1:** Quality assessment of diffusion data. (a) The average absolute motion observed within the patient data is comparable to that of controls. (b) The total outliers within the patient data are not that different from controls even though they are slightly higher in the patients’ data.


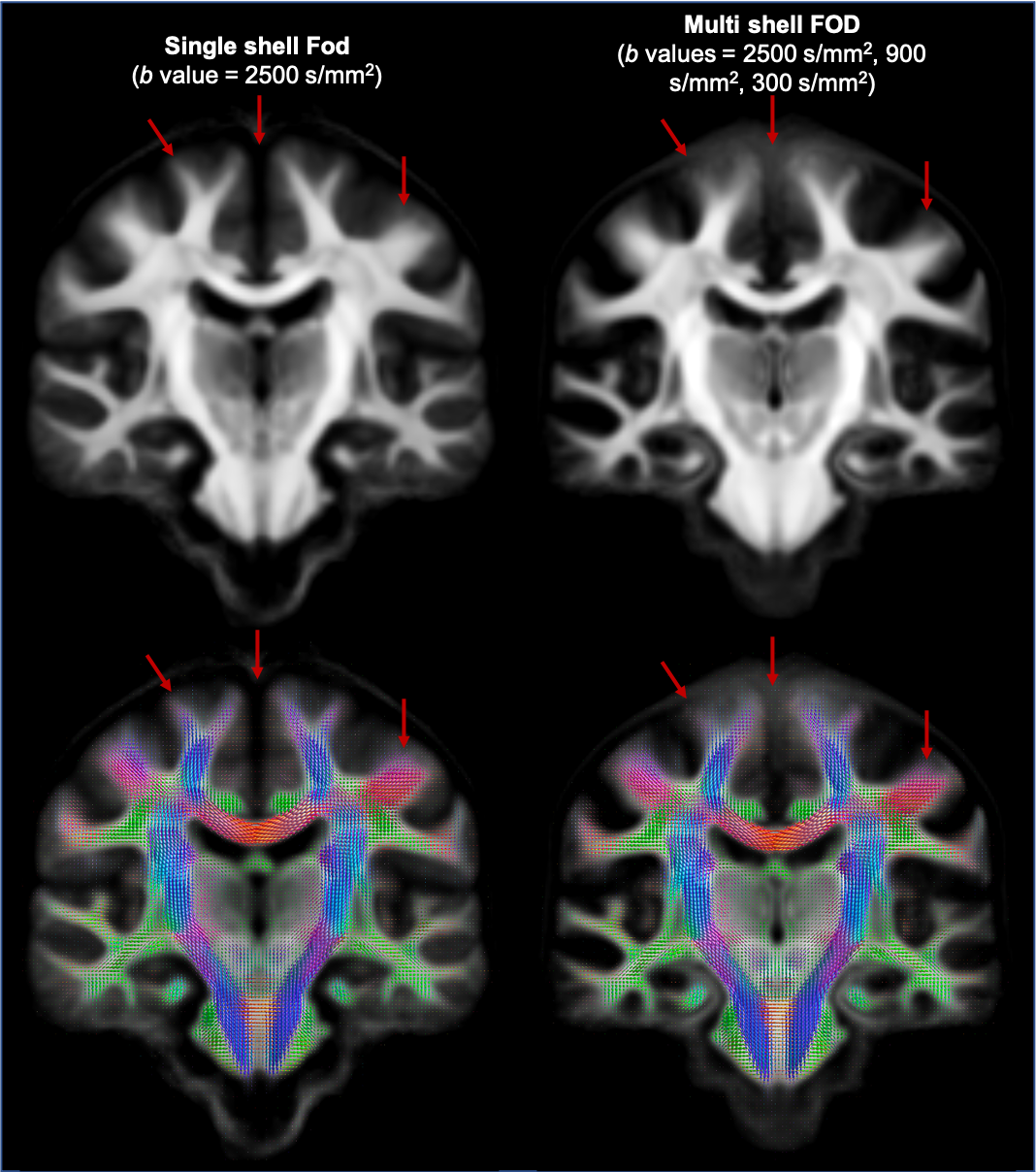


**Supplementary Figure 2:** FOD estimates from single shell and multi-shell data. Abandoning the low *b* values (900 s/mm^2^ and 300 s/mm^2^) and only using the highest *b* value (2500 s/mm2) led to improved estimation of the FOD in some regions.
